# Exosomal miRNAs in the plasma of *Cynoglossus semilaevis* infected with *Vibrio harveyi*: Pleiotropic regulators and potential biomarkers involved in inflammatory and immune responses

**DOI:** 10.3389/fimmu.2022.949670

**Published:** 2022-08-18

**Authors:** Tengfei Zhu, Ming Kong, Chen Li, Changwei Shao

**Affiliations:** ^1^ Key Lab of Sustainable Development of Marine Fisheries, Ministry of Agriculture and Rural Affairs, Yellow Sea Fisheries Research Institute, Chinese Academy of Fishery Sciences, Qingdao, China; ^2^ Laboratory for Marine Fisheries Science and Food Production Processes, Pilot National Laboratory for Marine Science and Technology, Qingdao, China; ^3^ College of Marine Life Science, Ocean University of China, Qingdao, China; ^4^ Key Laboratory of Maricultural Organism Disease Control, Ministry of Agriculture and Rural Affair, Qingdao Key Laboratory of Mariculture Epidemiology and Biosecurity, Yellow Sea Fisheries Research Institute, Chinese Academy of Fishery Sciences, Qingdao, China

**Keywords:** exosomes, miRNAs, inflammatory and immune responses, biomarker, teleost fish, IL-6

## Abstract

Exosomes are a class of extracellular vesicles with diameters ranging from ~50 to 150 nm. Incorporating diverse biological substances and being present extensively in biofluids, exosomes are involved in intracellular communication in various physiological and pathological processes and emerging as promising biomarkers for the prognosis and diagnosis of many diseases. Accumulating evidence shows that exosomes also play important roles in the inflammatory and immune responses to bacterial infection. However, the study of exosomes in teleost fish remains scarce. In the present study, we focused on the exosomal microRNAs (miRNAs) in the plasma of Chinese tongue sole (*Cynoglossus semilaevis*) in response to *Vibrio harveyi* infection. After bacterial challenge, the plasma was sampled at both the early (6 and 16 h) and late stages. (48, 72, and 96 h) of infection, followed by exosome isolation and exosomal miRNA sequencing. Results showed that the expression profile of 85 exosomal miRNAs was significantly different among the control, early-, and late-infection groups. The predictive genes targeted by exosomal miRNAs were extensively involved in various inflammatory and immune processes by Gene Ontology (GO) and Kyoto Encyclopedia of Genes and Genomes (KEGG) enrichment analyses, suggesting that a series of processes were regulated by exosomal miRNAs in the plasma, including the pathogen invasion and recognition and the activation and regulation of signaling pathways related to cytokine production. Moreover, the spleen was found to be a preference for exosome enrichment and the underlying mechanism of interleukin-6 production regulated by ZC3H12A, ARID5A, and exosomal ssa-miR-146a-5p were probably present in Chinese tongue sole. Additionally, the enhanced levels of ssa-miR-146a-5p and nbr-miR-731 in plasma exosomes and the spleen of the infection groups were identified, indicating their application as biomarkers in favor of the prognosis and diagnosis of *V. harveyi* infection in Chinese tongue sole. Therefore, the collective results in the present study indicated the pleiotropic roles of exosomal miRNAs in the regulation of inflammatory and immune responses and their potential utilization as biomarkers in teleost fish.

## Introduction

Exosomes are a subset of extracellular vesicles (EVs) with a size range from ~50 to 150 nm (~100 nm on average) (1) and secreted by most cell types and species ranging from prokaryotes to higher eukaryotes and plants (2). Their origination from endosomes distinguishes exosomes from other types of EVs (3). After endosome formation *via* endocytosis or budding from the trans-Golgi network and endoplasmic reticulum (ER), the intraluminal vesicles (ILVs, the future exosomes) were generated by endosome inward invagination, resulting in the formation of multivesicular bodies (MVBs). There are two fates for the MVBs; one is to be degraded by fusing with lysosomes or autophagosomes, and the other is to secrete exosomes by fusing with the plasma membrane (4, 5). In accordance with their formation process, exosomes contain various substances originating from either the outside microenvironment or the inside cellular products, including proteins, lipids, nucleic acids, amino acids, and other metabolites (6, 7). After budding from the donor cells, exosomes with their cargoes are transported in various biofluids (2) and ultimately taken up by recipient cells nearby or at a distance, accomplishing their missions as mediators in intercellular communication (1, 5). Accumulating research has shown that exosomes are involved in a variety of physiological and pathological processes including immune responses, reproduction and development, cancer progression, and so forth (8). Owing to their advantages in stability and accessibility, exosomes also show great potential as biomarkers in the diagnosis and prognosis of cancer and diseases of multiple organs including the central nervous system, liver, kidney, lung and arteries (9), and infection and inflammation (10).

With regard to the pathogenic infection, a number of studies have shown that exosomes derived from the hosts as well as the pathogens played important roles in the regulation of immune responses, either with a beneficial effect to activate the host immune responses or with a harmful effect to spread infection and suppress immune responses by delivering virulence compounds (10–12). For example, Bhatnagar S. et al. found that macrophages infected with intracellular pathogens releasing the exosomes containing pathogen-associated molecular patterns (PAMPs) could stimulate the proinflammatory response in a Toll-like receptor (TLR) and myeloid differentiation factor 88 (MyD88)–dependent manner and stimulated the production of TNF-α and IL-12 production *in vivo* and *in vitro* (13). Exosomes derived from the dendritic cell expressed a glycoconjugate that is cross-reactive with the capsular polysaccharide of *Streptococcus pneumoniae* type 14, inducing the humoral immune response *in vivo* (14). However, the anthrax lethal toxin, a major *Bacillus anthracis* virulence factor, encompassed in the exosomes could escape from the neutralization by the immune system and transmit to uninfected cells (15).

Among the various molecules incorporated in exosomes, microRNAs (miRNAs), a class of small non-coding RNAs with ~22 nt in length, have attracted much attention because they serve as negative posttranscriptional regulators of gene expression (16). By binding to the 3’ untranslated region (3’UTR) of mRNA, miRNAs lead to mRNA degradation or translational repression. An miRNA may have the ability to regulate more than 200 genes, and each mRNA may contain multiple binding sites for the same or different miRNAs (17, 18). Furthermore, some studies showed that there were several sorting mechanisms guiding specific intracellular miRNAs to enter exosomes, resulting in distinct miRNA profiling under different physiological conditions (19–22). In mammals, a number of studies found that exosomal miRNAs provided a novel form of intercellular communication during pathological inflammation and immune responses. The intercellular communication mediated by exosomal miRNAs connected various types of immune or non-immune cells together, playing vital roles in the regulation of inflammatory or immune responses (23–27). Additionally, exosomal miRNAs were found to be the ligands of TLRs; thereby, they can activate the innate immune system (28–30).

The study of exosomes in teleost fish is important from a phylogenetic standpoint; however, only a few studies on exosomal proteins and miRNAs have investigated their implications in inflammatory and immune responses. For instance, 35 unique proteins, such as the proteasome subunit, leukocyte cell–derived chemotaxin 2, cathelicidin antimicrobial peptide, protein S100, granulins, major histocompatibility complex (MHC) class I and II histocompatibility antigens, Toll-like leucine-rich repeat protein, and cathelicidin-derived antimicrobial, were identified in the EVs from the serum of *Salmo salar* infected with *Piscirickettsia salmonis* (31). There were 19 miRNAs differently enriched in the EVs released from the “monocyte-like” head kidney leukocytes (HKLs) in comparison with the “macrophage-like” HKLs in Atlantic salmon. Among them, ssa-miR-146a-5p, ssa-miR-155-5p, and ssa-miR-731-5p were significantly upregulated (*P*<0.001) in the “macrophage-like” HKLs, suggesting their important roles in HKL differentiation (32). A total of 10 viral proteins were identified in the exosomes derived from the kidney cells of the grass carp infected by the grass carp reovirus (GCRV), suggesting that exosomes may play important roles during GCRV infection (33).

In order to provide insights into the roles of piscine exosomes in the immune response to bacteria infection, the present study used the Chinese tongue sole (*C. semilaevis*) as a model, which is an economically important marine fish and the whole genome of which has been reported (34). After bacterial challenge by the intraperitoneal injection of *Vibrio harveyi*, exosomes were isolated from the plasma by the ultracentrifugation (UC) method and identified by measuring size, morphology, and marker proteins. Then, we analyzed the exosomal miRNAs in early and late phases of infection and uninfected groups by next-generation sequencing and found different miRNA patterns among them. Functional analyses *via* the GO term and KEGG pathway enrichment suggested that a series of inflammatory and immune responses to bacterial infection were potentially affected by exosomal miRNAs, including endocytosis, cell adhesion, signal transduction, the regulation of transcription, protein phosphorylation, mitogen-activated protein kinase (MAPK) signaling pathway, oxidation–reduction process, metabolic process, ion transport, and metal ion binding. The spleen was proven to be a major organ for plasma exosome biodistribution after *In Vivo* Imaging System (IVIS) analysis. The increased levels of ssa-miR-146a-5p and nbr-miR-731 in both exosomes and the spleen of the infection group indicated the miRNA exchange between exosomes and spleen, suggesting their roles as promising biomarkers. Moreover, we proposed a possible model of interleukin-6 (IL-6) regulation engaged by exosomal ssa-miR-146a-5p, exemplifying one of the functions fulfilled by exosomal miRNAs in response to bacteria infection.

## Materials and methods

### Fish and bacterial challenge

Healthy *C. semilaevis* (body weight, 433.7 ± 69.1 g; length, 41.0 ± 2.6 cm) used in the present study were purchased from Laizhou Mingbo Aquatic Co., Ltd., in Yantai, China. Forty fish were distributed into eight plastic tanks with five fish per tank (100-L tank) randomly. All fish were acclimatized to the experimental conditions at 22°C with aeration for 1 week prior to the *Vibrio harveyi* challenge. The *V. harveyi* strain was obtained from the Key Laboratory for Sustainable Development of Marine Fisheries (Ministry of Agriculture, Yellow Sea Fisheries Research Institute, Chinese Academy of Fishery Sciences, Qingdao, China) and cultured in 2216E broth at 28°C. Finally, the *V. harveyi* with a concentration adjusted to 3 × 10^6^ colony-forming units (CFU)/ml in 0.01 M phosphate-buffered saline (PBS) was harvested and used for the *V. harveyi* challenge experiment. After the acclimation, 30 fish in five tanks were challenged by the intraperitoneal injection of *V. harveyi* with a volume, depending on the fish weight (0.5 μl/g), which is named the infection group. The 10 fish in the other two tanks were injected intraperitoneally with PBS (0.5 μl/g) as sham infection and named the control group. The parameters of water quality were monitored daily during the experiment.

### Sampling of plasma and spleen

**Figure 1:**
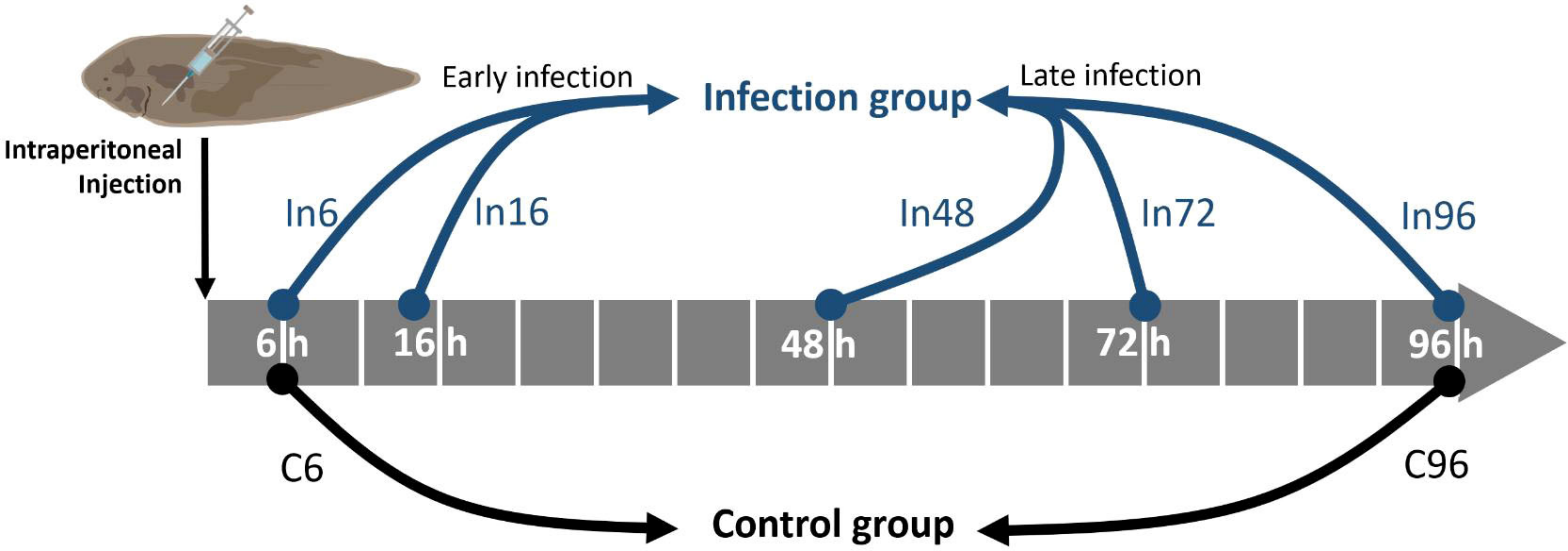
Schematic illustration of the sampling time points for bacterial challenge experiment. After the intraperitoneal injection of *Vibrio harveyi*, the blood and spleen were sampled at 6, 16, 48, 72, and 96 h, which were named as In6, In16, In48, In72, and In96, respectively. Correspondingly, the control groups were sampled at 6 and 96 h after the intraperitoneal injection of PBS in order to prevent the stresses caused by time, which were named as C6 and C96, respectively. The five groups with bacterial infection were further divided into early-infection (In6 and In16) and late-infection (In48, In72, and In96) groups.

Fish were euthanized by benzocaine (E1501, Sigma-Aldrich, Darmstadt, Germany) dissolved in 100% ethanol with a final concentration of 50 mg/L in seawater. Body weight and length were measured. Three individual fish from the infected group were sampled at five time points (6, 16, 48, 72, and 96 h after bacteria injection) and they were named as In6, In16, In48, In72, and In96, respectively, of which the In6 and In16 were regarded as the early-infection groups and In48, In72, and In96 were regarded as the late-infection groups. Furthermore, three individuals from the control group were sampled at the beginning and the end of the sampling time points (6 and 96 h after PBS injection) in order to prevent the stresses caused by time and they were named as C6 and C96, respectively (**Figure 1**). As plasma was more favorable for extracellular vesicle (EV) isolation than serum that may contain platelet-derived EVs during blood clotting (35), we used Ethylene Diamine Tetraacetic Acid (EDTA) as an ideal anticoagulant that was also compatible for downstream RNA-related analysis. Specifically, blood was drawn from the caudal vein by syringes rinsed with EDTA into the tubes with EDTA drops (~10 ul) and inverted immediately several times before storing at 4°C. The blood samples were then centrifuged at 400 × *g* for 10 min at 4°C to remove cells and platelets. The plasma samples were collected from the resultant supernatant and stored at -80°C for exosome isolation. Spleen was dissected and immediately frozen in liquid nitrogen and kept at -80°C for gene and miRNA expression analyses.

### Exosome isolation from the plasma

Exosomes from the plasma were isolated by the differential UC method depending on the protocol specialized for viscous fluids (36), and some modifications were made to be adapted for the present study. The plasma samples were first diluted with an equal volume of PBS, followed by the centrifugation of the dilution at 2,000 × g for 30 min. The resultant supernatant was collected carefully without inhaling the contaminates at the bottom and transferred to fresh tubes, followed by centrifugation at 12,000 × g for 45 min. The supernatant was then transferred carefully to the ultracentrifuge tube (326819, Beckman Coulter) without contamination, followed by UC at 120,000 × g for 2 h with a rotor of SW55Ti (Optima XPN-100, Beckman, Beckman Coulter, Brea, CA, US). The precipitation was collected and suspended in PBS and then filtered through a 0.22-µm filter membrane (SLGP033RB, Millipore, Billerica, MA, US). After the aggregates and particles of large sizes were removed, the filtrate was collected for a second UC at 120,000 × g for 70 min. The resultant pellet was resuspended in PBS for a third UC at 120,000 × g for 70 min. Eventually, the pellet was resuspended in a moderate amount of PBS and stored at -80°C for exosome analysis. All the centrifugation steps were performed at 4°C.

### Exosome characterization

#### Total protein measurement and Western blotting

After the exosomes were disintegrated by RIPA, the amount of total proteins in exosomes was measured by the bicinchoninic acid (BCA) Kit according to the manufacturer’s instruction (PC0020, Solarbio). Total protein was extracted using RIPA protein lysis buffer containing 1-mM phenylmethanesulfonylfluoride (PMSF), and then, 20-µg protein was subjected to sodium dodecyl sulfate polyacrylamide gel electrophoresis (SDS-PAGE) and transferred to a polyvinylidene fluoride membrane (Millipore). After blocking in 5% defatted milk for 2 h at room temperature, the membrane was incubated with specific primary antibodies at 4°C overnight. The antibodies against the following proteins were used for Western blot analysis: CD63 (1:1,000 dilution, Abcam), CD81 (1:500 dilution, Abclonal, Wuhan, China) and HSP70 (1:1,000 dilution, Abcam). After being washed three times with TBST (TBS containing 0.1% Tween-20), the membrane was incubated with a horseradish peroxidase–conjugated secondary antibody (1:4,000 dilution, Abcam) for 1 h at room temperature. Following membrane washing, the membrane was visualized by chemiluminescent reagents (Thermo Fisher, Waltham, MA, US).

#### Transmission electron microscopy

Approximately 10 µl of exosome samples were dropped on a parafilm. A 200-mesh carbon/formvar coated grid was floated on the sample droplet for 10 min, followed by eliminating the excess fluid from the grid with a filter paper. The grid was transferred to a 15-µl drop of distilled water on the parafilm for 1 min. The grid was then placed reversely on a drop of 2.5% glutaraldehyde for 10 min, followed by excess fluid elimination. Then, negative staining was conducted by floating grid on a 10-μl drop of 2% uranyl acetate for 1 min. The grid was dried and examined using a transmission electron microscope operated at 120 kV (Hitachi, Tokyo, Japan).

#### Nano-flow cytometry

Approximately 100-nm orange FluoSpheres of known concentrations was used to calibrate the sample flow rate. The sample size and concentration were detected by simultaneously collecting the side scatter and orange fluorescence of individual exosomes by a nano-flow cytometry instrument (Xiamen, China). The focused laser beam illuminated completely the sample stream. Ultrapure water was used as the sheath fluid, and the distribution histogram was collected in 1 min.

### RNA library construction, sequencing, and obtaining microRNAs

A total of 21 exosome samples from control and infection groups were used for RNA library construction. Total RNAs were extracted from with a TRIzol Reagent (Invitrogen, Waltham, MA, USA). Small RNA libraries were constructed and sequenced with TruSeq Small RNA Donor Prep Kits (Illumina Inc., San Diego, CA, USA) by Lianchuan Biotechnological Company Ltd. (Hangzhou, China). Raw reads were subjected to an in-house program, ACGT101-miR (LC Sciences, Houston, TX USA) to remove adapter dimers, junk, low complexity, common RNA families (rRNA, tRNA, snRNA, snoRNA), and repeats. Subsequently, unique sequences with length in 18~26 nucleotides were mapped to specific species precursors in miRBase 22.0 by Basic Local Alignment Search Tool (BLAST) search to identify known miRNAs and novel 3p- and 5p-derived miRNAs.

### The prediction of microRNA target genes and the analysis of differentially expressed microRNAs

TargetScan (v5.0) and Miranda (v3.3a) were used to predict the miRNA-binding sites in the target genes. Finally, the data predicted by both algorithms were combined and the overlaps were calculated. The GO terms and KEGG pathway of the miRNA targets were also annotated. The differential expression of miRNAs based on normalized deep-sequencing counts was analyzed by selectively using the Fisher exact test, chi-squared 2 × 2 test, chi-squared nXn test, Student’s t-test, or ANOVA based on the experiment design. The significance threshold was set to be 0.01 and 0.05 in each test.

### Determination of plasma exosome biodistribution *in vivo* by microinjection and IVIS

Approximately 200 µl of exosomes from the control groups were mixed and incubated with 1-µM DiR (Invitrogen, USA) for 30 min at room temperature. Free DiR was then removed from DiR-labeled exosomes by using Amicon centrifugal filter devices (Millipore, USA). After fish anesthesia with benzocaine, the DiR-labeled exosomes were injected into three healthy fish (average weight, 1.2 g) at the posterior dorsal aorta by a microinjection system (WPI PV820, USA) with a dose of 8 µl/fish. After 6 h, the fluorescence images of live fish body from both dorsal and ventral sides and major organs after dissection were recorded by IVIS (Wilber NEWTON 7.0, France).

### Quantitative real-time PCR

The expression of miRNAs in exosomes and spleens and the expression of genes in spleens were detected by qRT-PCR. Total RNA in exosomes and spleens were extracted by an miRNeasy Serum/Plasma Advanced Kit (Qiagen, Hilden, Germany) and TRIzol (Invitrogen, USA), respectively. The miRcute Plus miRNA First-Strand cDNA kit (Tiangen, Beijing, China) and FastKing RT kit (Tiangen, Beijing, China) were used to synthesize the cDNA of miRNAs and mRNAs, respectively. qPCR was implemented in 384-well plates with a LightCycler 480 II qRT-PCR system (Roche Diagnostics, Mannheim, Germany). The MiRcute Plus miRNA qPCR kit (SYBR Green) (Tiangen, China) was used for the detection of miRNA expression with the following program: predenaturation at 95°C for 15 min, followed by 45 cycles of denaturation at 94°C for 20 s and annealing and extension at 60°C for 34 s. The Talent qPCR PreMix (SYBR Green) kit (Tiangen, China) was used for the detection of gene expression with the following program: predenaturation at 95°C for 3 min, followed by 40 cycles of denaturation at 95°C for 5 s and annealing and extension at 60°C for 15 s. Melting curve analysis was conducted at the end of the programs. Each PCR assay included replicate samples (the duplicate of reverse transcription and PCR amplification) and negative controls (RT- and cDNA-free samples, respectively). U6 and *β-actin* were used for the normalization of the miRNA and gene expression, respectively. The relative quantification of expression was determined using the E-Method from the LightCycler 480 software (version SW 1.5; Roche Diagnostics). The primers used for the RT-qPCR are listed in **Table 1**.

**Table 1:** Sequences of the primers used for gene and microRNA (miRNA) expression analysis by quantitative real-time PCR.

miRNA/Gene	Forward Primers	Reverse Primers
oni-let-7e	GCGGGTGAGGTAGTAGATTGAATAGTT	/
hhi-miR-15a_R-1	GGTAGCAGCACGGAATGGTTTGT	/
oni-miR-16b	GCGTAGCAGCACGTAAATATTGGAG	/
ssa-miR-146a-5p	GCGTGAGAACTGAATTCCATAGATGG	/
nbr-miR-731	GATGACACGTTTTCTCCCGGATT	/
b3gnt2	TCCGAACGCTCAAACCTAGT	GCCCACACAGTGTTAAAGCA
zc3h12a	GCAGAGATCAGCAGAAACGG	CTCCTGAGACATGTTGGGGT
zbtb16	CTTCTGTGGTTTTGTCCCCG	TGCTTCATCGTGTTGTGCAA
irf7	AAGCACAACTCGAGGAAGGA	TGAAACGCACAGACAGGTTG
il-6	GGTCTCAACCCTCAAAGCCA	ACGCACTCAAGAGTCCATGG
arid5a	CCACCCGGAAGCTAACCAAT	ACATCAGCTGAGACTGTGGC
U6	GGAATGATACAGAGAAGATTAGC	TGGAACACTTCTCGAATTTGCG
β-actin	CCCTGGAGAAGAGCTACGAG	GTACCTCCAGACAGCACAGT

## Results

### Characterization of exosomes from plasma

**Figure 2:**
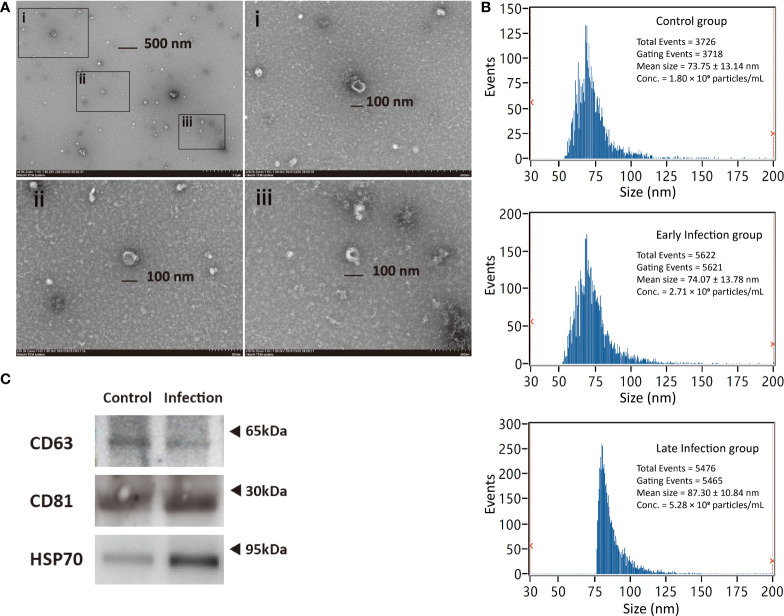
Characterization of exosomes from *Cynoglosussus semilaevis* plasma. **(A)** Exosomes morphology viewed by transmission electron microscopy with both wide-field image (Top left) and close-up images (i-iii). i–iii denote the close-up images of exosomes with a diameter of ~100 nm at three different zones of the wild-field image. **(C)** Particle size distribution and concentration in the control (C6 and C96), early- (IN6 and IN16), and late-infection (IN48, IN72, IN96) groups analyzed by Nano-FCM. **(B)** Western blotting examination of exosome biomarkers (CD63, CD81, and HSP70) in control (C6 and C96) and infection (IN6, IN16, IN48, IN72, and IN96) groups.

According to the recommendations from Minimal information for studies of extracellular vesicles 2018 (MISEV2018) (37), both general and single vesicle characterization were implemented in order to identify the exosomes from the plasma of *Cynoglosussus semilaevis*. The images at high resolution provided by transmission electron microscopy (TEM) showed that the diameter of most vesicles was approximately 100 nm and most vesicles were cup shaped in morphology, which was the typical shape of exosomes observed under TEM (**Figure 2A**). Then, we measured the size and concentration of the particles by Nano-FCM, the diameter of these particles were distributed in a range from 50 to 125 nm, with a mean size of approximately 74 nm in the control and early infection groups and approximately 87 nm in the late- infection group, which was in agreement with the size range of typical exosomes (38) (**Figure 2B**). The concentrations of the particles detected by Nano-FCM in control, early- and late-infection groups were 1.80×10^9^, 2.71×10^9^, and 5.28×10^8^ particles/ml, respectively (**Figure 2B**). Moreover, CD63, CD81 (transmembrane proteins), and HSP70 (cytosolic proteins) all showed positive results in both control and infection groups, indicating the presence of exosomes in terms of marker proteins for their general characterization (**Figure 2C**). Meanwhile, the protein concentration of the particles measured by the BCA method showed that there was no significant difference among the seven groups; all were within a range from 0.54 to 1.63 mg/ml **(** **Supplementary Figure 1** **)**. These results confirmed that the vesicles isolated from the plasma were basically exosomes, and they were qualified for further sequencing analysis and subsequent experiments.

### Construction of microRNA library

The sequencing results of the 21 (n=3) exosomal samples showed that a total of 42814031, 39394198, 41213395, 46265057, 40818120, 42503600, and 46730479 raw reads were obtained from the libraries of C6, C96, In6, In16, In48, In72, and In96, respectively. After removing the sequences of adaptor, low quality, and length less than 18 nt (ACGT101-miR program), we obtained 30099032 (70.3%), 28365699 (72.0%), 29663422 (72.0%), 28910789 (62.5%), 30982326 (75.9%), 28959784 (68.1%), and 32591836 (69.7%) clean reads in each group, which represented 3017648 (46.9%), 2879876 (47.4%), 2880615 (48.9%), 3918742 (53.8%), 2655429 (49.7%), 3378360 (49.5%), and 3371643 (46.8%) unique reads, respectively (**Supplementary File 1**). Except for the unmapped reads, the other reads were mapped to protein-coding RNAs, non-coding RNAs, and repetitive DNAs. Specifically, in the percentage of the total mapped reads, mRNA (24.31%–54.48%), miRNA (13.86%–32.39%), rRNA (11.75%–26.77%), and tRNA (8.66%–25.96%) were the four dominating RNAs in all groups, while snoRNA (0.10%–0.21%), snRNA (0.06%–0.81%), and repetitive DNA (0.18%–0.52%) were in relatively trace quantities (**Supplementary File 1**). After mapping the unique sequences with length in 18~26 nucleotides to miRBase 22.0 by BLAST, a total of 865 known miRNAs and 394 novel miRNAs were identified in the present study. Specifically, we identified 641, 649, 610, 647, 706, 597, and 635 known miRNAs and 179, 136, 174, 163, 177, 123, and 147 novel miRNAs in C6, C96, In6, In16, In48, In72, and In96, respectively. Collectively, we identified 725 and 815 known miRNAs (with 675 in overlap) and 219 and 334 novel miRNAs (with 150 in overlap) in the control and infection groups, respectively, and 680 and 775 known miRNAs (with 640 in overlap) and 225 and 257 novel miRNAs (with 148 in overlap) in the early- and late- infection groups, respectively (**Supplementary File 1**). After counting the miRNA length, we found that 22-nt miRNAs were the highest in number and the second highest miRNAs were those with 23 nt (**Supplementary Figure 2**).

### Different exosomal microRNA patterns in control, early-, and late-infection groups

**Figure 3:**
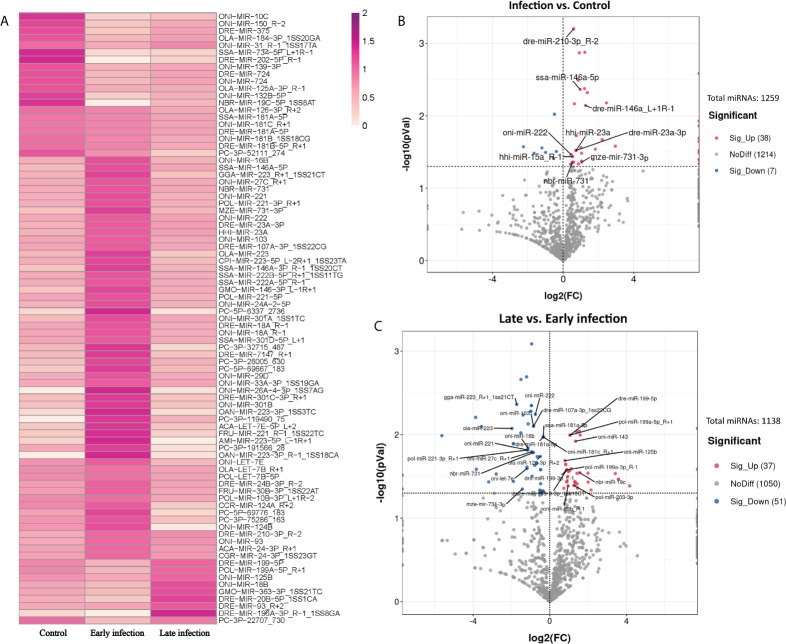
Exosomal miRNAs in the plasma with distinguished levels in control, early and late infection groups. **(A)** Heatmap showing the levels of 85 exosomal miRNAs with significant differences in control, early and late infection groups (*P* < 0.05) (Dark red: higher level; Light red: lower level). **(B)** Volcano plot showing the levels of 45 exosomal miRNAs with significant differences between control and infection groups (*P* < 0.05). (Red points: higher level in infection group; Blue points: lower level in infection group; Grey points: no significant differences between control and infection groups; Points with labels: high expressed miRNAs). **(C)** Volcano plot showing the levels of 88 exosomal miRNAs with significant differences between early and late infection groups (*P* < 0.05). (Red points: higher level in late infection group; Blue points: lower level in late infection group; Grey points: no significant differences between early and late infection groups; Points with labels: high expressed miRNAs).

In order to identify the exosomal miRNAs featuring the infection either in early or late stages, three groups, that is, the control group (C6 and C96), the early-infection group (In6 and In16), and the late infection group (In48, In72, and In96), were established and compared with each other. In total, 85 exosomal miRNAs were significantly different among the three groups using the normalization methods with a *P*-value < 0.05 after ANOVA analysis (**Figure 3A**), which manifested that the exosomal miRNAs in the plasma were affected by the infection and could be changed with the phases of infection. To further screen those exosomal miRNAs specifically affected by the infection, we combined the infection groups at early and late stages to one group and compared it with the control group. According to the result showed in the volcano plot, there were 45 exosomal miRNAs with significantly different levels between the infection group and control group; specifically, the levels of 38 miRNAs were significantly upregulated and 7 were significantly downregulated in the infection group (*P* < 0.05) (**Figure 3B**).

According to the normalized copy numbers of miRNAs in each samples, the miRNAs could be classified into three categories. Those less than 10 in all samples were regarded as low-expressed miRNAs, those more than 10 in at least one sample but less than the average in all samples were regarded as middle-expressed miRNAs, and those more than the average in at least one sample were regarded as highly expressed miRNAs. Given that the miRNAs with higher expression levels were more likely to be detectable in practice and perform biological functions, nine miRNAs with higher expression involved in the response to infection were selected as candidates for further analysis (**Figure 3B** and **Supplementary File 2**).

As the profiles of exosomal miRNAs in the plasma could also be changed with the stages of infection, we further compared the early-infection group (In6 and In16) with the late-infection group (In48, In72, and In96). The result showed that the level of 88 miRNAs were significantly different between early- and late-infection groups with 37 miRNAs significantly higher expressed and 51 miRNAs lower expressed in the late stage of infection (*P* < 0.05) (**Figure 3C**). Similarly, 26 differentially expressed miRNAs were selected as candidates for further analysis. Among them, 10 miRNAs showed higher levels in the late stage of infection in comparison with those in the early stage of infection, while 16 miRNAs showed lower levels in the late stage of infection in comparison with those in the early stage of infection (**Figure 3C** and **Supplementary File 2**). In the 26 miRNAs, oni-miR-222, nbr-miR-731, and mze-miR-731-3p were downregulated in the late stage of infection and they were also found to be significantly upregulated in the infection group in comparison with the control group.

### Prediction of microRNA target genes and their GO and KEGG enrichment analyses

**Figure 4:**
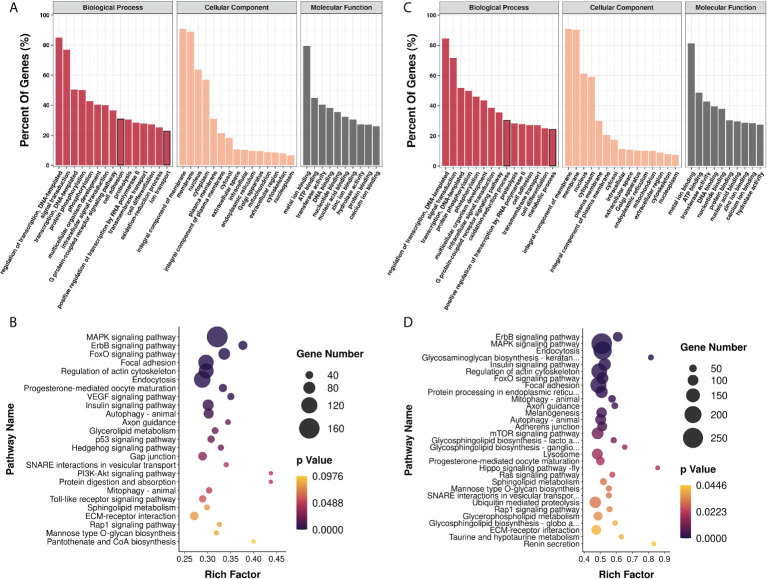
Functional analysis of genes targeted by exosomal miRNA candidates by GO and KEGG enrichment. GO **(A)** and KEGG **(B)** enrichment analysis of 5193 genes predicted to be targeted by high level miRNAs with significant differences between control and infection groups; GO **(C)** and KEGG **(D)** enrichment analysis of 9266 genes predicted to be targeted by high level miRNAs with significant differences between early and late infection groups.

We then proceeded to analyze the potential biological function of the selected miRNAs. The miRNA candidates comprising the 9 miRNAs affected by infection and 26 miRNAs changed with the infection stages were subjected to target gene prediction and then GO and KEGG pathway enrichment analyses, respectively. As a result, for the nine exosomal miRNAs responding to the infection condition, a total of 5,193 genes were predicted to be the target genes by TargetScan and Miranda methods and they were found to be associated with 5,646 GO term categories and 273 KEGG pathways (**Supplementary File 3**). Most genes were involved in the regulation of transcription, signal transduction, transcription, protein phosphorylation, intracellular signal transduction, the G protein–coupled receptor signaling pathway, cell adhesion, proteolysis, and so on. In terms of the cellular component and molecular function, the target genes were mostly related to the integral component of membrane and metal ion binding, respectively. As for KEGG enrichment analysis, most genes were enriched in the pathways related to MAPK signaling, endocytosis, focal adhesion, and so on (**Figures 4A, B**). Meanwhile, there were a total of 9266 genes predicted to be the targets of the 26 exosomal miRNAs which changed with the infection stages. These target genes were associated with 7413 GO term categories and 295 KEGG pathways (**Supplementary File 3**). The results of GO enrichment showed that they were almost similar to the findings above. It should be noted that the oxidation-reduction process and metabolic process ranked higher than cell adhesion and ion transport, respectively, whose rank were inversed in the results above. Similar to the findings of KEGG enrichment analysis above, most genes were enriched in the pathways involved in MAPK signaling, endocytosis, focal adhesion, and so on (**Figures 4C, D**).

### Investigation of microRNA candidates and their target genes involved in inflammatory and immune responses in exosomes and spleen

**Figure 5:**
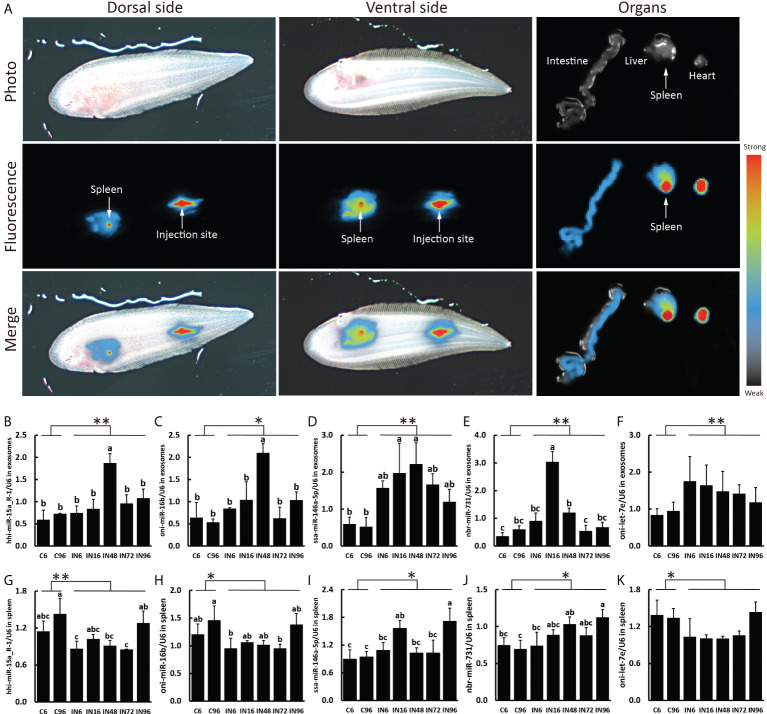
Plasma exosomes biodistribution and expressions of miRNA candidates in exosomes and spleen. **(A)** Representative images of the plasma exosome biodistribution in live fish and their organs at 6 h after Dorsal aorta microinjection of DiR-labeled exosomes derived from plasma. Images of dorsal and ventral sides and organs were captured by IVIS. The expressions of hhi-miR-15a_R-1 **(B)**, oni-miR-16b **(C)**, ssa-miR-146a-5p **(D)**, nbr-miR-731 **(E)**, oni-let-7e **(F)** in exosomes and the expressions of hhi-miR-15a_R-1 **(G)**, oni-miR-16b **(H)**, ssa-miR-146a-5p **(I)**, nbr-miR-731 **(J)**, oni-let-7e **(K)** in spleen. Expression values were normalized by U6. Values are means (n=3) with their standard deviations represented by vertical bars. ^a,b,c^ Mean values with unlike letters were significantly different (*P* < 0.05, One-way analysis of variance, Tukey’s Test). **P < 0.01; *P < 0.05 versus the controls.

Next, we decided to focus on the miRNAs that were predicted to target the genes involved in inflammatory and immune responses by examining the GO terms and KEGG pathways and consulting the previous bibliographies. Five pairs of miRNA-transcript with a TargetScan score more than 80 as well as Miranda energy less than -40 were selected for further analyses (**Table 2**). Since the spleen is a large secondary lymphoid organ performing a wide range of immunologic functions and a primary site filtering and monitoring the blood (39, 40), it is of great significance to analyze the candidate miRNAs and their target genes in the spleen. In order to validate the relationship between plasma exosomes and the spleen, we detected the biodistribution of plasma exosomes in fish by microinjection and IVIS. As expected, the enrichment of plasma exosomes in spleen was manifested in the strong fluorescent signal of the spleen, indicating that the spleen could be a primary organ affected by plasma exosomes and, accordingly, the levels of miRNAs and genes in the spleen could be closely related to the miRNAs from plasma exosomes (**Figure 5A**). Therefore, on one hand, we detected the levels of hhi-miR-15a_R-1, oni-miR-16b, ssa-miR-146a-5p, nbr-miR-731, and oni-let-7e in the plasma exosomes to validate the sequencing results; on the other hand, we detected their levels in the spleen to investigate if there was a relationship between miRNAs in plasma exosomes and spleen. The results showed that the levels of hhi-miR-15a_R-1 and oni-miR-16b in exosomes were the highest at 48 h after infection (**Figures 5B,C**). The exosomal ssa-miR-146a-5p exhibited high levels in all groups of infected fish, with significantly higher levels at 16 and 48 h after infection than those at 6 and 96 h without infection (**Figure 5D**). The exosomal nbr-miR-731 also showed significantly higher levels at 16 and 48 h after infection than that at 6 h without infection (**Figure 5E**). However, no significant differences were found for the levels of exosomal oni-let-7e in the seven groups (**Figure 5F**). The levels of all five exosomal miRNAs were significantly higher in the infected fish than those in non-infected fish. The higher levels of exosomal hhi-miR-15a_R-1, ssa-miR-146a-5p, and nbr-miR-731 were consistent with their sequencing results showing more copies in the infection groups (**Figures 5B-F**).

**Table 2:** Five pairs of candidate miRNAs and their target genes involved in inflammatory and immune responses selected by TargetScan and Miranda methods.

Gene	Gene Annotation	Gene ID	Transcript ID	miRNA ID	TargetScan score	Miranda Energy
*irf7*	interferon regulatory factor 7 isoform X1	103380393	NM_001294228.1	nbr-miR-731	87	-47.8
*b3gnt2*	N-acetyllactosaminide beta-1,3-N-acetylglucosaminyltransferase 2	103387698	XM_008322454.3	hhi-miR-15a_R-1	80	-66.12
*b3gnt2*	N-acetyllactosaminide beta-1,3-N-acetylglucosaminyltransferase 2	103387698	XM_008322454.3	oni-miR-16b	80	-54.05
*zc3h12a*	endoribonuclease ZC3H12A	103387896	XM_008322701.3	ssa-miR-146a-5p	86	-57.04
*zbtb16*	zinc finger and BTB domain-containing protein 16	103395382	XM_008333081.3	oni-let-7e	95	-51.61

The expressions of hhi-miR-15a_R-1 and oni-miR-16b in the spleen showed similar patterns that they were both higher expressed in the fish without infection and the fish at 96 h after infection (**Figures 5G, H**). The expression of ssa-miR-146a-5p in the spleen was significantly increased at 16 and 96 h after infection compared with that at 6 and 96 h without infection (**Figure 5I**). The spleen at 96 h after infection expressed the highest level of nbr-miR-731 as well, which is significantly higher than those without infection (**Figure 5J**). Similar to the level of exosomal oni-let-7e in plasma, oni-let-7e in the spleen showed no significant differences in all seven groups (**Figure 5K**). Of note is that the expressions of both ssa-miR-146a-5p and nbr-miR-731 in spleen were significantly higher in infected fish than those in non-infected fish, which were consistent with the results in exosomes. However, there were much higher expressions of hhi-miR-15a_R-1, oni-miR-16b, and oni-let-7e in the spleen of non-infected fish than those of infected fish, which were reversed to the results in exosomes (**Figures 5G-K**).

**Figure 6:**
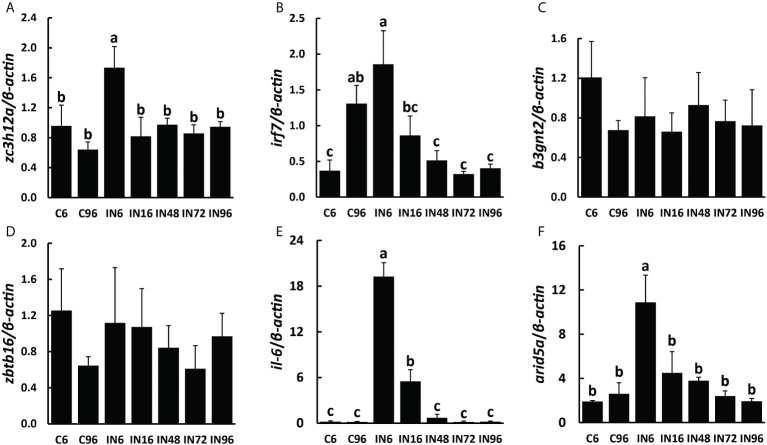
Expressions of target genes involved in inflammatory and immune responses in spleen. The expressions of *zc3h12a* **(A)**, *irf7* **(B)**, *b3gnt2* **(C)**, *zbtb16* **(D),** *il-6* **(E)** and *arid5a* **(F)** in spleen. ^a,b,c^ Mean values with unlike letters were significantly different (*P* < 0.05, One-way analysis of variance, Tukey’s Test).

Subsequently, we analyzed the expressions of inflammation and immune- related genes targeted by the five candidate miRNAs in the spleen, including *b3gnt2*, *zbtb16*, *zc3h12a*, and *irf7*. Interestingly, the results showed that the expression of *zc3h12a* predicted to be targeted by ssa-miR-146a-5p was significantly promoted in the spleen of fish at 6 h after infection and then reduced to the same level as that in the control groups (**Figure 6A**). The *irf7* predicted to be targeted by nbr-miR-731 was also higher expressed in the spleen of fish at 6 h after infection, but then its expression dropped gradually to a lower level at 16 h. Finally, the expression of *irf7* at 48, 72, and 96 h after infection reached the level similar to that at 6 h without infection; however, the expression of *irf7* at 96 h without infection was also promoted (**Figure 6B**). Although the expressions of *b3gnt2* and *zbtb16* in the spleen showed some fluctuations, there were no significant differences in the seven groups (**Figures 6C, D**).

### The expressions of *il-6* regulated by ZC3H12A and ARID5A

According to the foregoing results, the levels of ssa-miR-146a-5p and nbr-miR-731 in both exosomes and the spleen were significantly higher in infected fish than those in non-infected fish, indicating the possibility that there could be exchanges of ssa-miR-146a-5p and nbr-miR-731 between plasma exosomes and the spleen during infection. Moreover, the splenic expressions of *zc3h12a* and *irf7* predicted to be targeted by ssa-miR-146a-5p and nbr-miR-731, respectively, were also significantly affected by infection; particularly, the *zc3h12a* showed significantly higher expression at 6 h after infection than the control. We then tended to investigate the IL-6 known to be degraded by ZC3H12A and ARID5A known to inhibit the IL-6 degradation by counteracting ZC3H12A (41). The result showed that the expression of *il-6* was significantly promoted at 6 h after infection and then reduced to a lower level at 16 h after infection; the level at 48, 72, and 96 h after infection were almost the same low as that at 6 and 96 h without infection (**Figure 6E**). Similar to the findings of IL-6, the expression of *arid5a* was also significantly increased at 6 h after infection (**Figure 6F**).

## Discussion

To acquire sufficient exosomes with less contaminations from the tissues is a prerequisite for their subsequent function analyses. As plasma is the physiological medium of EVs in the blood and serum and may contain platelet-derived EVs released during clotting (35), we chose plasma instead of serum for exosomal miRNA analyses. UC is acknowledged as the current gold-standard technique with the advantage in low protein contamination (38). Therefore, we used the UC method for exosome isolation and made some modifications to adapt for the present plasma condition, depending on the protocols made by Théry C. et al. (36), including diluting the plasma before centrifugation and increasing the speed and lengths of centrifugations. After the size was analyzed by Nano-FCM, the diameter distribution of the particles was from 50 to 150 nm and averaged approximately 80 nm, suggesting that large extracellular vesicles and protein aggregates were mostly eliminated. With the cup-shaped morphology displayed by TEM and both of the transmembrane (CD63, CD81) and cytosolic (HSP70) proteins certified by WB, these particles purified from the plasma in the present study were basically identified as exosomes. However, when inspecting the images of TEM, we also found some small white and round particles that could possibly be the very low density lipoproteins (VLDLs, 30–80 nm) as it was reported that lipoproteins were often isolated together with extracellular vesicles from the plasma samples (42). Although lipoproteins may be the inevitable contaminants in the isolated exosomes, we still conducted the subsequent exosomal miRNA analyses because miRNAs were virtually not reported to be present in the VLDL except for the high-density lipoproteins (HDLs) (43).

As noted above, exosomal miRNAs are informative to inflammatory and immune responses and could be promising biomarkers for the prognosis and diagnosis of diseases. Meanwhile, the next-generation sequencing, one of the most powerful techniques for the comprehensive profiling of the nucleic acids, has been utilized to investigate the miRNAs in exosomes derived from either the biological fluids (44–46) or the cell-conditioned media (22, 47, 48). These studies showed that there were big differences not only in the content and profiling of miRNAs but also the other nucleic cargos including mRNA, rRNA, and tRNA, which may be caused by the different cell types, methods for exosome and RNA isolation, and sequencing platforms (49). In the present study, the exosomal miRNA reads in each group accounted for 13.86% to 32.39% of all mapped reads, and a total of 1,259 miRNAs (865 known miRNAs and 394 novel miRNAs) were identified. The percentage was much less than the one shown in the study of exosomal miRNAs derived from human plasma that accounted for 76.20% of all mapped reads and detected 593 known miRNAs (44). Another study also showed that the read percentage of exosomal miRNAs derived from the plasma of *C. semilaevis* was 78.30% and 85.72% (50), which was higher than the percentage in the present study as well. However, a previous study investigated that the miRNAs in exosomes derived from the mucus of *C. semilaevis* infected with *V. harveyi* showed that the clean reads mapped to the miRbase was only 0.06% and 146 and 251 known miRNAs were identified in the infected and control groups, respectively (51). The variations of the miRNA content in exosomes from these studies may be attributed to both technical and biological factors. Of note, the percentage of miRNAs was not dominated in the plasma exosomes in the present study, suggesting that other nucleic acids incorporated in the exosomes may respond to bacterial infection. Since it was reported that the intracellular TLRs can bind both double- and single-stranded nucleic acids from viruses and bacteria (52), there were possibilities that nucleic acids aside from miRNA incorporated in the exosomes may bind to the TLRs for immune response activation in the present study.

**Figure 7:**
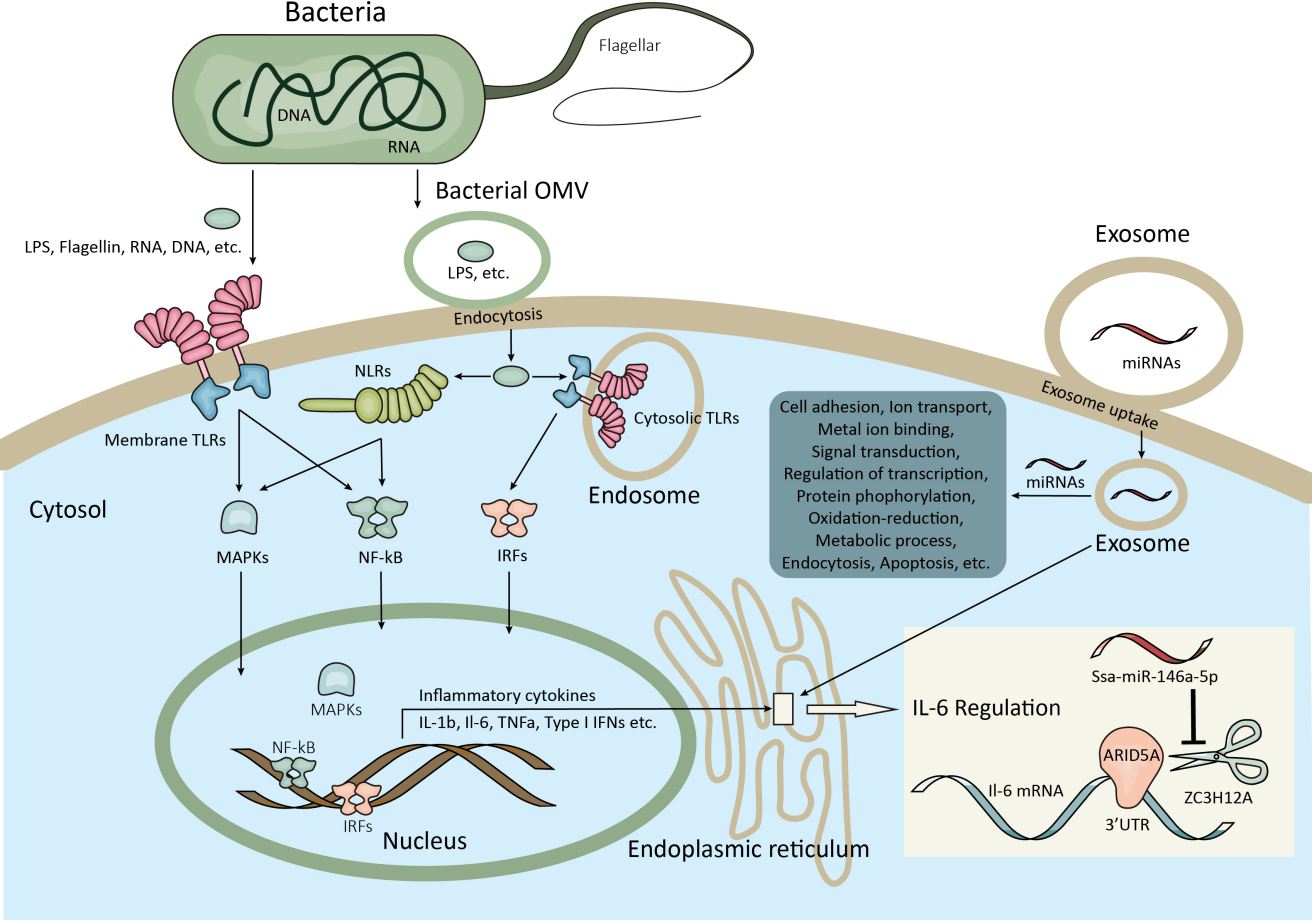
Schematic illustration of the inflammatory and immune responses potentially regulated by exosomal miRNAs in the present study. The bacterial virulence factors such as flagellar, proteases, etc. facilitate the adhesion and invasion of bacteria to the host cell. Meanwhile, the bacterial virulence factors including flagellin, RNA, DNA, LPS, etc. are recognized by the host membrane PRRs (e.g. TLRs) or transported by bacterial OMVs into the host cytosol by endocytosis and then recognized by cytosolic PRRs (e.g. TLRs, NLRs). After pathogen recognition, signaling pathways involved in the regulation of inflammatory and immune responses were activated at both transcriptional and posttranscriptional levels (e.g. NF-κB, IRFs, MAPK), resulting the homeostasis of cytokine production. Moreover, the bacteria are able to compete for metal ions and affect the metabolic process of the host cell. The above-mentioned processes are potentially regulated by exosomal miRNAs after exosome uptake by endocytosis or other ways. For example, ZC3H12A can degrade IL-6 mRNA by binding to its 3’UTR on ER, which are counteracted by ARID5A, while the ZC3H12A mRNA could be inhibited by ssa-miR-146a-5p from the exosomes which have access to the ER. LPS, Lipopolysaccharide; PRRs, Pattern recognition receptors; TLRs, Toll-like receptors; OMVs, Outer membrane vesicles; NLRs, Domain-like receptors; NF-κB, Nuclear factor−κB; IRFs, Interferon-​regulatory factors; MAPK, Mitogen-activated protein kinase; IL-6, Interleukin-6; 3’UTR, 3’ Untranslated region; ER, Endoplasmic reticulum.

As expected, the heat map of the miRNA pattern showed significant differences among the control, early-infection and late-infection groups, suggesting that the 85 miRNAs may operate as regulators during different stages of infection and could be potential biomarkers indicating the *V. harveyi* infection and further the stages of infection. Since the miRNAs with more copies in the exosomes were more accessible and informative in terms of biomarkers and biology, we focused on a total of 35 miRNAs with more copies in the plasma exosomes, which showed significant differences between the control and infection groups and the early- and late-infection groups, respectively. According to the results of GO and KEGG enrichment analyses, the functions of the target genes were mainly involved in the regulation of transcription, signal transduction, protein phosphorylation, cell adhesion, proteolysis, and so on in terms of GO and the MAPK signaling pathway, endocytosis, and focal adhesion in terms of KEGG. Of note, the cell adhesion ranked higher regarding infection while the oxidation–reduction process stood out regarding infection stages. These results seem to be comprehensible when we considered the processes of pathogen invasion and recognition and the correspondingly inflammatory and immune responses in animals. As we know, *V. harveyi* is a Gram-negative bacterium, belonging to family *Vibrionaceae* of class *Gammaproteobacteria* (53). Studies have shown that *V. harveyi* is a serious bacterial pathogen of invertebrates, fish, and mammals (54–58). Further, the pathogenicity mechanisms of *V. harveyi* in fish may be attributed to many virulence factors and their interactions, such as flagellar, biofilms, hemolysins, proteases, lipases, ferric chelators, and lipopolysaccharide (LPS) (59–62). The processes of host invasion by pathogenic bacteria were conducted by these virulence factors step by step (63). The flagellar motility is responsible for the initial interaction of a bacterium with the surface of the host cell, followed by the adhesion to the external and mucosal surfaces of the host. It was reported that the flagellar played important roles in the virulence of *V. harveyi* (64), although the detailed information remains uninvestigated. There were genes targeted by exosomal miRNAs enriched in cell adhesion and focal adhesion in the present study, particularly more enriched in the infection groups, indicating that the process of pathogen invasion was potentially regulated by exosomal miRNAs in the present study. After attaching to the host cell surface, the bacterium secreted types of lytic enzymes including hemolysin, proteases, and lipases in order to enter the host cell and get access to the nutrients, resulting in host tissue damage and apoptosis. It was found that the *V. harveyi* hemolysin caused death to the erythrocytes and gill cells of the Japanese flounder *via* the caspase activation pathway (65); similar results were found in the fibroblast of sea bream (66). This may be the reason why some target genes were enriched in proteolysis and the regulation of apoptotic processes in the present study, suggesting that cell damage and death were regulated by exosomal miRNAs as well. When arriving in the interior of the host cell, the bacterium has to develop a system to acquire metal ions that are indispensable for bacterium survival but in a conjugated form in the host cell. Indeed, it was found that the ferric chelators in *V. harveyi* are responsible for the iron acquisition and transport (67). Likewise, the present study also suggested that the genes involved in ion transport and metal ion binding were regulated by the exosomal miRNAs. From the perspective of the host, it is known that the pattern recognition receptors (PRRs) located in the host cell membrane or cytoplasm are able to recognize the conserved microbial products that are known as the pathogen-associated molecular patterns (PAMPs), stimulating innate immune response and host defense (68, 69). The PRRs that were first identified and have been extensively studied are TLRs that are able to detect various components of the bacteria, including LPS, flagellin, lipoproteins, dsRNA, and rRNA (69). A previous study showed that TLR2 present in the Chinese tongue sole was a potential TLR recognizing a wide range of bacteria, including *V. harveyi*, and stimulated the expression of genes involved in the MyD88-dependent signaling pathway, such as *myd88*, *irak4*, *traf6*, *tak1*, *nf-kB/p105*, *tnf-a*, *il-1b*, and *il-8* (70). Moreover, nucleotide binding oligomerization domain–like receptors (NLRs) were another type of PRRs that were reported to be reactive to peptidoglycan (PGN)-derived molecules, bacterial RNA, endogenous danger signals, LPS, and so on in the cytoplasm, activating proinflammatory cytokines *via* the transcription factor nuclear factor–κB (NF-κB) signaling pathway and apoptosis *via* caspase1 (71–73). In Chinese tongue sole, a recent investigation showed that NLRs encoded by 29 genes were expressed ubiquitously in various tissues and some of them were sensitive to *V. harveyi* infection, especially those in the intestine, kidney, liver, and spleen (74). Therefore, the above-mentioned processes involved a series of signal transduction pathways and the regulation of transcription, which were also the major enriched functions by most target genes in the present study. After PAMP recognition through distinct PRRs, the stimulated innate immune cells induce proinflammatory responses (the production of cytokines and chemokines) and recruit additional immune cells. It is well known that the proinflammatory responses are tightly controlled at transcriptional (e.g., NF-κB, signal transducers and activators of transcription, and interferon-​regulatory factors) and post-transcriptional (MAPK) levels in order to prevent tissue damage from excessive inflammatory responses (75, 76). Again, the present results that most genes were enriched in the regulation of transcription and protein phosphorylation suggested that these processes were regulated by exosomal miRNAs in the plasma. It was also reported that the glucose utilization and metabolic changes occurred in the long-term TLR-stimulated cells (77), suggesting that the oxidation–reduction process and metabolic process enriched particularly in the late stage of infection were regulated by exosomal miRNAs in the plasma as well. Additionally, it is worth noting that Gram-negative bacteria were found to secrete outer membrane vesicles (OMVs) for LPS delivery into the host cell cytosol by endocytosis, resulting in the activation of cell death or cytokine production *via* caspase-11 (78), which provided a novel perspective under bacteria infection. The entrance of exosomes and OMVs into cells are both associated to endocytosis, which is another process enriched by most genes by KEGG analyses in the present study, suggesting one more process regulated by exosomal miRNAs in the present study. In summary, the functional analyses of the target genes in the present study suggested that the miRNAs delivered by plasma exosomes were extensively involved in the regulation of a series of inflammatory and immune processes including the pathogen invasion and its competition for nutrients and energy with the host and the host responses from pathogen recognition to cytokine production in Chinese tongue sole infected with *V. harveyi* (**Figure 7**).

In order to exemplify the specific genes targeted by exosomal miRNAs involved in inflammatory and immune responses, we screened the genes targeted by candidate miRNAs with TargetScan and Miranda methods and investigated their expressions in the spleen, which is the largest secondary lymphoid organ with extensive functions involved in inflammatory and immune responses and blood filtering and monitoring (39, 40). The biodistribution of plasma exosomes by IVIS analysis confirmed their enrichment in the fish spleen, suggesting the necessity to focus on the spleen for further functional exploration of plasma exosomes. ZC3H12A, also known as regnase-1 and mcpip1, was an RNase critical for maintaining immune homeostasis in mammals by degrading mRNAs involved in inflammation. Studies in mice showed that ZC3H12A promoted the mRNA degradation of IL-6 and IL-12p40 by binding to their 3’UTR (79). Furthermore, Mino, T. et al. revealed that ZC3H12A functioned in the ribosome and ER with a preference for degrading the inflammation-related mRNAs in the early phase of inflammation (80). In the present study, *zc3h12a* was predicted to be the potential target gene of ssa-miR-146a-5p. Interestingly, results showed that the expression of *zc3h12a* was significantly promoted at 6 h after infection, but the level of ssa-miR-146a-5p in the spleen and exosomes showed a delayed promotion at 16 h after infection, suggesting that the expression of *zc3h12a* in spleen at 16 h was inhibited by ssa-miR-146-5p, which may be delivered from the plasma exosomes since ssa-miR-146-5p in exosomes and the spleen synchronized to high levels at 16 h and the ZC3H12A located on the ER was more approachable to exosomes, depending on the mechanisms of exosome uptake (81). Accordingly, we further investigated the splenic expression of *il-6* reported to be targeted by ZC3H12A (79) and the AT-rich interactive domain containing protein 5a (ARID5A), which was able to counteract the ZC3H12A degradation of IL-6 mRNA by competing for the 3’UTR (82). Results showed that the expression of *il-6* was elevated significantly in the early phase of infection with the highest expression at 6 h after infection. Interestingly, the expression of *arid5a* exhibited a similar pattern to *il-6*. As we know, IL-6 was synthesized at the initial stage of inflammation with pleiotropic effects on inflammation and immune response, such as inducing the production of acute- phase proteins and regulating the serum level of iron and zinc; however, the dysregulation of IL-6 production leads to various diseases in mammals (41). The higher expression of *il-6* at 6 and 16 h in the present study indicated that the important roles of IL-6 involved in the early stage of inflammation and immune response were also present in the Chinese tongue sole. Meanwhile, the expression of *il-6* seems to be controlled by ZC3H12A and ARID5A, which were both increasingly expressed at 6 h as well. Therefore, a hypothesis was made that IL-6 may play important roles in early inflammatory and immune responses in the Chinese tongue sole infected with *V. harveyi*, and the expression of *il-6* was finely regulated by ZC3H12A and ARID5A in the spleen. This regulation may be affected by the exosomal ssa-miR-146a-5p from the plasma by targeting ZC3H12A mRNA (**Figure 7**). However, further studies are required to confirm this hypothesis.

Furthermore, the levels of ssa-miR-146a-5p and nbr-miR-731 in both exosomes and the spleen were significantly enhanced in the infection group, which were consistent with the results of miRNA sequencing, suggesting that these miRNAs could be potential biomarkers. Interestingly, the study of miRNAs released from the EV of HKLs in Atlantic salmon also indicated the involvement of ssa-miR-146a and ssa-miR-731 in macrophage differentiation and immune responses (32). Of note, interferon regulatory factor 7 (*irf7*), the predicted target gene of nbr-miR-731 and a regulator of type I IFN production, apoptosis, and immune cell differentiation (83), showed higher expression in the spleen at 6 h after infection as well, suggesting another potential mechanism regulated by exosomal nbr-miR-731 in the present study. Therefore, the regulatory mechanisms of both miR-146a and miR-731 warrant future investigations.

In conclusion, we investigated the profiles of exosomal miRNAs in the plasma of Chinese tongue sole at the early and late stages of infection with *V. harveyi*, suggesting that a wide range of genes involved in the regulation of inflammatory and immune responses were potentially regulated by exosomal miRNAs. The hypothesis of IL-6 regulation mediated by ZC3H12A, ARID5A, and plasma exosomal ssa-miR-146a-5p proposed a novel perspective for further research on the role of exosomes in intracellular communication for cytokine homeostasis. Moreover, the practical application of ssa-miR-146a-5p and nbr-miR-731 in plasma exosomes as biomarkers would benefit the prognosis and diagnosis of bacterial infection in Chinese tongue sole.

## Data availability statement

The data presented in the study are deposited in the NCBI Sequence Read Archive (SRA) repository, accession number PRJNA832244.

## Ethics statement

The animal study was reviewed and approved by Animal Care and Use Committee at the Yellow Sea Fisheries Research Institute, Chinese Academy of Fishery Sciences.

## Author contributions

Conceptualization: TZ and CS; Data curation: TZ; Formal analysis: TZ; Investigation: TZ; Methodology: TZ, CL, and MK; Validation: TZ, CS, MK, and CL; Writing - original draft: TZ; Writing - review and editing: TZ, CS, MK, and CL. All authors contributed to the article and approved the submitted version.

## Funding

This work was supported by the National Key R&D Program of China (2018YFD0900301); the National Nature Science Foundation of China (32002384); the AoShan Talents Cultivation Program Supported by Qingdao National Laboratory for Marine Science and Technology (2017ASTCP-ES06); the Taishan Scholar Project Fund of Shandong of China; the National Ten-Thousands Talents Special Support Program; the Central Public-interest Scientific Institution Basal Research Fund, CAFS (No.2020TD19) and the Qingdao Postdoctoral Applied Research Projects.

## Conflict of interest

The authors declare that the research was conducted in the absence of any commercial or financial relationships that could be construed as a potential conflict of interest.

The reviewer HC declared a shared affiliation with the author MK to the handling editor at time of review.

## Publisher’s note

All claims expressed in this article are solely those of the authors and do not necessarily represent those of their affiliated organizations, or those of the publisher, the editors and the reviewers. Any product that may be evaluated in this article, or claim that may be made by its manufacturer, is not guaranteed or endorsed by the publisher.

## Supplementary material

Supplementary Figure 1 Total protein concentration of exosomes measured by BCA method.

Supplementary Figure 2 microRNA length distribution.

Supplementary File 1 Construction of microRNA library.

Supplementary File 2 microRNA candidates with significantly different levels between infection and non-infection, early- and late-infection groups.

Supplementary File 3 Predicted target genes of the microRNA candidates and their GO and KEGG analyses.
