# Supplementary figures and images for: Exosomal miRNAs in the plasma of Cynoglossus semilaevis infected with Vibrio harveyi: Pleiotropic regulators and potential biomarkers involved in inflammatory and immune responses
Source: Front Immunol. 2022 Aug 18;13:949670. doi: 10.3389/fimmu.2022.949670 (PMC9433998; doi:10.3389/fimmu.2022.949670)

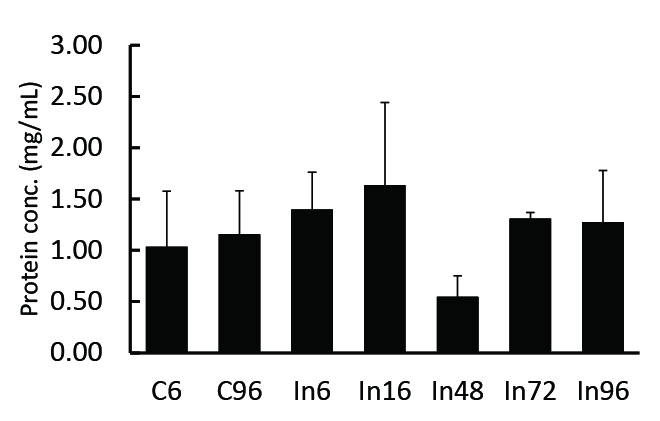

Supplement: Supplementary Figure 1 — Total protein concentration of exosomes measured by BCA method. [file Image_1.jpeg]

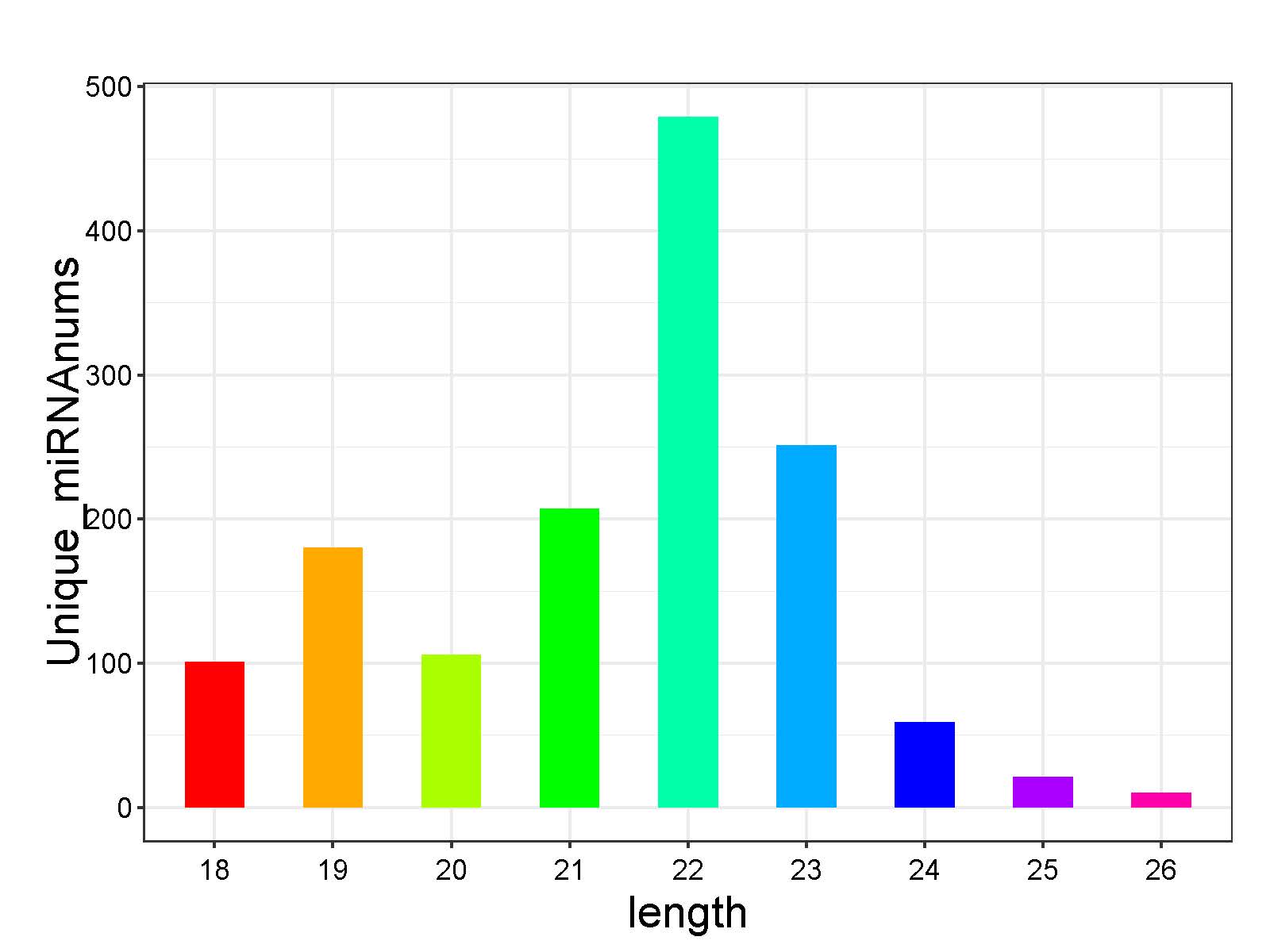

Supplement: Supplementary Figure 2 — microRNA length distribution. [file Image_2.jpeg]
